# Radiation-shielding glass with tailored Al-doped zinc oxide (AZO) coatings for durable space photovoltaic modules

**DOI:** 10.1039/d5ra06756a

**Published:** 2025-11-04

**Authors:** Dajeong Kim, Yerang Park, Hyun-Beom Shin, Ho Kwan Kang, Da Young Kwon, Jae-ik Han, Nochang Park, Yonghwan Lee

**Affiliations:** a Advanced Batteries Research Center, Korea Electronic Technology Institute (KETI) Seongnam-si Gyeonggi-do Korea ncpark@keti.re.kr ylee@keti.re.kr; b Nano Process Division, Korea Advanced Nanofab Center (KANC) Suwon-si Gyeonggi-do Korea

## Abstract

In space applications, photovoltaic cells, which are used to provide power and enable the self-operation of satellites, are exposed to various extreme stressors. Among these, electron radiation induces various defects in photovoltaic cells, thereby accelerating performance degradation. To mitigate radiation-induced damage, effective shielding methods must be integrated into the photovoltaic module. In this study, we investigated aluminum-doped zinc oxide (AZO) thin-film-coated quartz glass as a potential radiation-shielding cover glass for III–V multi-junction space photovoltaic cells. To enhance the shielding performance, two post-treatment methods, ultraviolet (UV) treatment and thermal annealing, were tested. It was observed that thermal annealing improved the crystallinity of the AZO film and enhanced its effective shielding against electron radiation. Under electron irradiation at 1.2 MeV with a fluence of 1 × 10^15^ to 3 × 10^15^ e^−^ cm^−2^, the thermally annealed AZO-coated glass demonstrated superior radiation-shielding performance compared to bare quartz glass. Consequently, when applied to our in-house-developed space photovoltaic modules incorporating a 4G32C III–V photovoltaic cell, they resulted in an improvement in efficiency degradation, from 4.18% to 2.37%, after electron irradiation at 1.2 MeV and 1 × 10^15^ e^−^ cm^−2^. These findings demonstrate that AZO thin films, when prepared with appropriate post-treatment, can serve as reliable radiation-shielding layers, offering significant potential enhancement to the long-term durability and operating stability of space photovoltaic modules.

## Abstract 2

Thermally annealed AZO-coated glass provides effective electron radiation shielding, significantly mitigating efficiency degradation in III–V space photovoltaic modules under electron irradiation.

## Graphical abstract

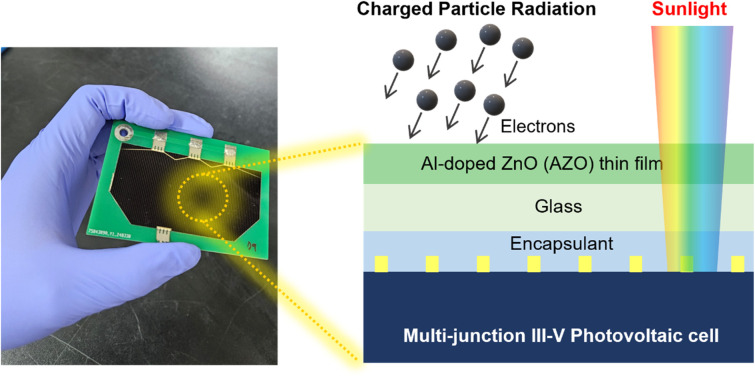

## Introduction

Since 1957, when the world's first artificial satellite was launched and placed into Earth's orbit, important milestones have been achieved that are crucial to space exploration. With increasing demand and the steady development of space technology, today more than 7000 artificial satellites are in orbit for various purposes, including communications, global positioning system (GPS), earth observation, and reconnaissance missions.^1^ These satellites require a reliable supply of electrical power, and, among available options, photovoltaic module systems can effectively provide continuous, stable power from sunlight without the need for an external recharging process.

The photovoltaic module system employed in spacecraft and satellites as an energy supply unit consists of photovoltaic cells as well as protective materials that shield them from external hazards. Unlike the conditions photovoltaic cells encounter on earth, stressors in the space environment, such as high-energy ionizing radiation (like electron, proton, neutron, heavy-ion gamma-ray, X-ray, UV), vacuum, space debris, micrometeorites, and extreme temperature cycles, are present.^2–4^ These stressors induce progressive degradation and, as photovoltaic cells accumulate defects in space, performance deterioration is accelerated. Importantly, shortening the operational lifetime of space photovoltaic cells significantly reduces the overall service life of satellites.

One of the most significant damaging factors of space satellite photovoltaic modules is charged particle radiation caused by trapped electrons.^5,6^ When electrons strike the photovoltaic cell, they can induce undesired defects in the photovoltaic cell. For instance, the striking particles modify the crystal structure of the photovoltaic cells by ionization or atomic displacement, resulting in vacancies, interstitials, or anti-sites.^3,7,8^ The formed defects might act like minority/majority-carrier traps, recombination centers, or generation centers, depending on the location of the defect's energy level in the bandgap. The defects formed by the charged particle irradiation lead to a reduction in the efficiency of the photovoltaic cells. To address this vulnerability and ensure longer lifetime and stable operation, an appropriate radiation hardening method has to be applied to the photovoltaic cells.

Encapsulating the front side of photovoltaic cells with a transparent radiation shield is a common approach to protect them from hazardous charged particles and other deleterious space environmental factors. The transparent radiation shield simultaneously transmits solar radiation in the visible range while effectively blocking hazardous charged particles. Glass is typically employed as a radiation shield because of its high thermal stability and excellent corrosion resistance.^2,9^ However, prolonged exposure to high doses of harmful radiation can generate undesired defect centers in the glass, leading to darkening and increased absorption in the visible range. Conventionally, cerium (Ce)-doped glass has been widely used to mitigate defect formation caused by the accumulation of ionizing radiation. In such glasses, coexisting Ce^3+^ and Ce^4+^ ions capture radiation-induced electrons and holes, suppressing the formation of the color centers that reduce transmittance in glass.^10–12^ Nevertheless, excessive Ce doping can also degrade optical transmittance in the visible range; thus, an appropriate level of Ce doping is required.

Another approach involves depositing a transparent shielding material on the glass substrate to enhance the shielding performance. Such thin film coatings on the glass substrate have been well developed to date, and numerous scalable coating methods have been established, enabling large-area deposition with high uniformity and offering high design flexibility. For instance, transparent conductive oxide (TCO)-coated glass is frequently employed as a transparent radiation shield for photovoltaic modules and spacecraft windows.^9,13,14^ In addition to shielding, the TCO coating mitigates the accumulation of space charges that generate local electric fields, thereby reducing the likelihood of electrostatic discharges (ESDs) through efficient charge dissipation.

In this study, we systematically investigated aluminum-doped zinc oxide (AZO), a representative TCO material widely used in applications, such as display and optoelectronic devices.^15,16^ In addition, AZO is also a good radiation-robust TCO candidate for perovskite photovoltaic cells.^17^ AZO layers can be readily synthesized and cost-effectively deposited onto glass substrates using facile coating methods. In addition, the material properties of AZO are known to vary significantly, depending on the post-treatment process. To systematically investigate AZO on glass, we analyzed the surface morphology, microstructure, and optical properties of AZO thin films coated on glass substrates after two different post-processes: ultraviolet (UV) exposure and thermal annealing.^18–21^ The electron radiation-shielding performance of AZO-coated glass was then evaluated for different post-annealing conditions. Finally, an optimized AZO-coated glass was applied as an efficient transparent shield on commercial space-grade III–V photovoltaic cells. The encapsulated modules with our optimized AZO-coated glass exhibited a significantly smaller degradation of efficiency compared to the reference modules encapsulated with bare glass, after exposure to electron irradiation at an energy of 1.2 MeV and a fluence of 3 × 10^15^ e^−^ cm^−2^.

## Results and discussion

**Fig. 1:**
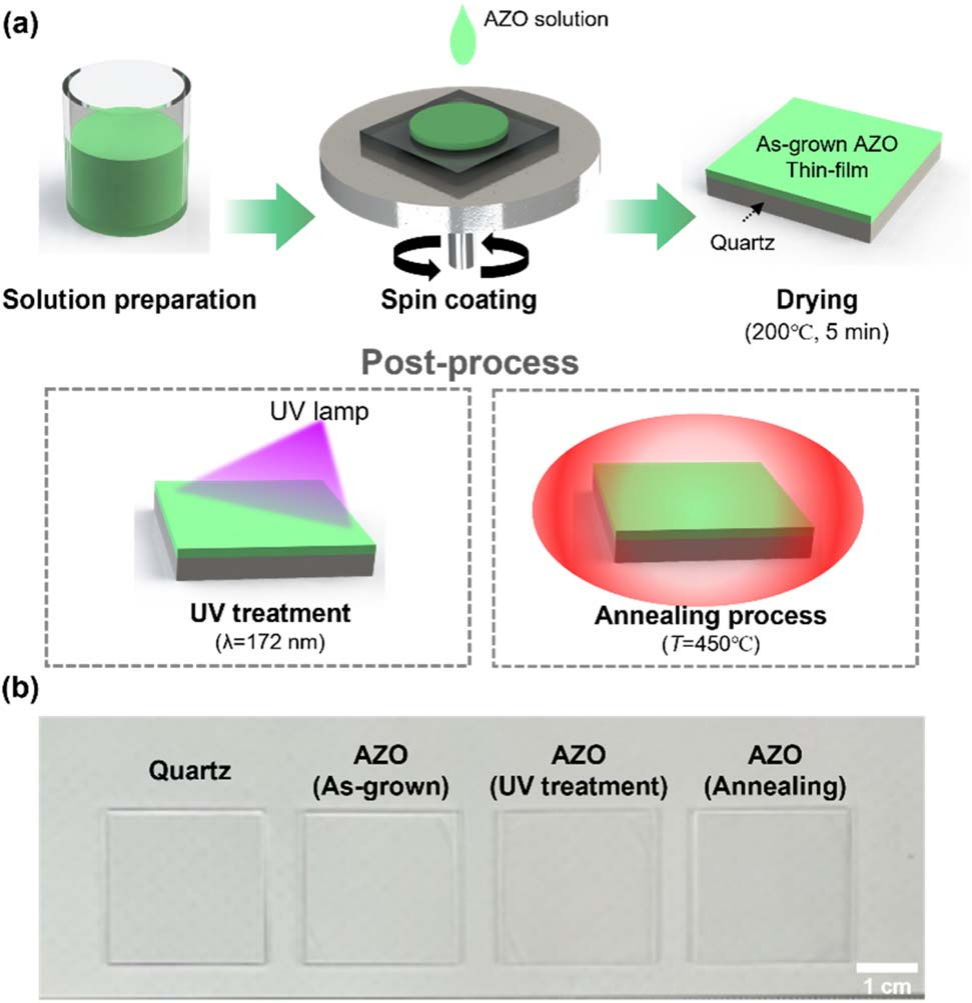
Process for fabricating radiation-resistant glass by coating AZO thin film. (a) Schematic illustration of the AZO spin coating and post-treatment processes on the quartz glass substrate. (b) Digital image of the bare quartz, the as-grown AZO, UV-treated AZO, and annealed AZO-coated quartz substrates.

Fig. 1a shows the fabrication process of the AZO thin film on quartz glass. The AZO precursor solution was synthesized using zinc acetate dihydrate [Zn(CH_3_COO)_2_·2H_2_O] and aluminum nitrate nonahydrate [Al(NO_3_)_3_·9H_2_O] as the sources of Zn and Al, respectively. The AZO solution was deposited on the glass substrate by a spin coating method.^22^ Quartz was used as the substrate to eliminate parasitic effects due to the substrate itself, ensuring that the radiation effects were solely evaluated on the AZO thin film layer.^17^ To modulate the microstructure of the AZO thin films, we employed two types of post-deposition processes on the AZO thin film: an ultraviolet (UV) process and a thermal annealing process in a conventional box furnace.

The two types of post-treatment methods were tested to form a good radiation-shielding cover glass for the space photovoltaic module. Because UV light has high energy, it can decompose organic groups in the precursor thin films. Carbon and oxygen atoms are volatilized to small molecules, while the M–O–M (metal–oxide–metal) network framework of metal and oxygen atoms is activated.^23^ In this way, the UV process can modify the conductivity, optical transmission, and surface morphology of various metal oxides. For example, Tseng *et al.* reported that a UV irradiation process enhanced the crystallinity and conductivity of AZO film.^19^ At the same time, post-annealing treatment is considered an effective way to modify intrinsic defects and improve optical/electrical properties. Tong *et al.* reported that annealing treatment *via* a furnace enhanced the optical transmission and conductivity of AZO thin film.^20^ To investigate further improvement of the radiation-shielding performance of AZO thin film on glass substrate, the efficacy of these two post-treatment methods was compared. Fig. 1b exhibits a digital image of the films on a glass substrate, showing the bare quartz, and the as-grown, UV-treated, and annealed AZO thin films on quartz glass.

**Fig. 2:**
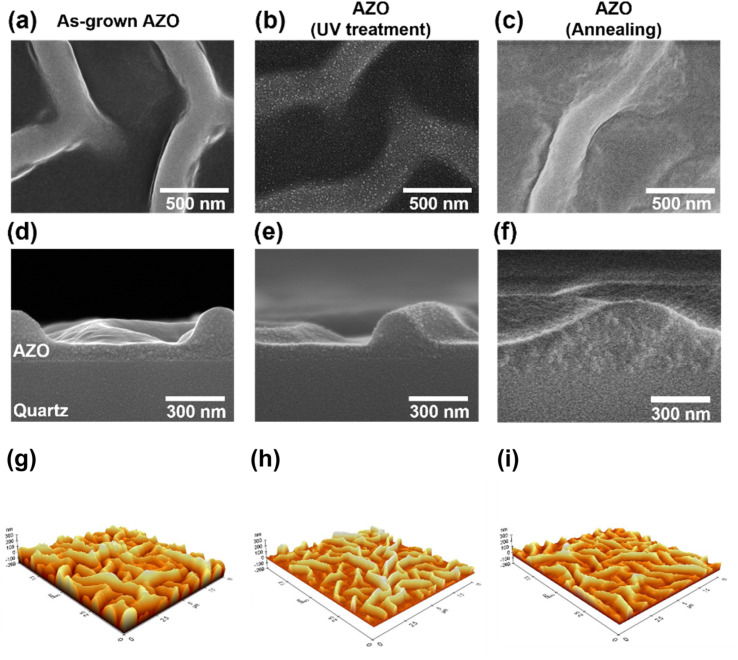
Characterization of the morphologies of AZO thin films on glass substrate. (a–c) Top view and (d–f) cross-sectional SEM images, and (g–i) AFM images of as-grown, UV-treated, and annealed AZO thin films on glass substrates.

Fig. 2a–f show top and cross-section scanning electron microscope (SEM) images of the AZO-coated glass (quartz) substrates. The as-grown AZO thin film, AZO thin film after UV treatment, and AZO thin film after the annealing process appear non-uniform with a wavy surface structure due to the formation of volumetric strain in the sol–gel-derived thin film during solvent evaporation (see Fig. 2a and d).^24,25^ Atomic force microscopy (AFM) analysis was also conducted on the AZO thin films to evaluate surface morphology. A slight reduction in surface roughness was observed after post-treatment. Specifically, the measured average roughness (*R*_a_) values were 55.24, 38.90, and 30.73 nm for the as-grown, UV-treated, and annealed films, respectively. We attribute this reduction in surface roughness to the relaxation of residual stress and the removal of residual organic compounds, which will be discussed in a later section.^21,26^

**Fig. 3:**
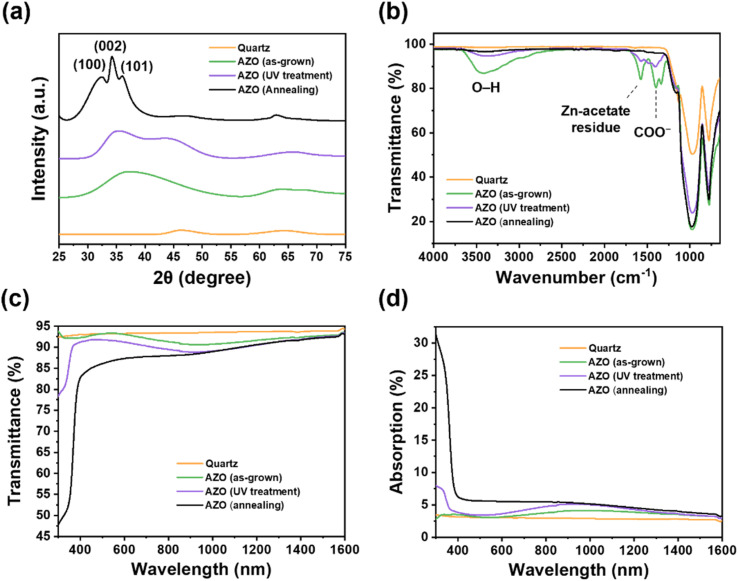
Characterization of the material properties of AZO thin films prepared using different post-treatment conditions. (a) XRD spectra, (b) FT-IR spectra, (c) optical transmission, and (d) absorption spectra of AZO thin films deposited on quartz substrate.

The crystallographic structure of the AZO thin films was investigated using the X-ray diffraction (XRD) method. Fig. 3a shows XRD patterns of the AZO thin films with different post-treatment processes. The XRD patterns of the as-grown and UV-treated AZO thin films denote an amorphous structure, whereas the thermally annealed AZO thin film exhibited a polycrystalline wurtzite hexagonal crystal structure.^12,21^ The XRD data of the annealed AZO thin film reveal peaks corresponding to the (100), (002), and (101) diffraction planes of the hexagonal ZnO crystal structure at 32.4°, 34.2°, and 36.1°, respectively.

The Fourier-transform infrared (FT-IR) spectroscopy spectra of the AZO thin film are displayed in Fig. 3b. In the FT-IR spectra, several noticeable chemical bonds are shown. The spectra of the as-grown AZO thin film show three main peaks: a broad O–H stretching band at 3405.4 cm^−1^, a symmetric COO^−^ stretching peak at 1397.6 cm^−1^, and a peak at 1576.5 cm^−1^. The broad O–H band is attributed to hydrogen-bonded hydroxyl groups, likely from residual water or alcohol. The 1397.6 cm^−1^ peak corresponds to the symmetric stretching of carboxylate groups from the zinc acetate precursor.^27^ The 1576.5 cm^−1^ peak is considered to come from intermediate oxide species or partially decomposed zinc acetate, rather than a typical Zn–O stretching vibration. These indicate that residual organic components from the precursor compounds (*e.g.*, zinc acetate dihydrate or monoethanolamine) remained on the as-grown AZO thin film. These peaks became weaker after the UV treatment process, but were not completely removed. However, after annealing, these peaks almost entirely disappeared. This means that the residual organic components in the precursor can be effectively removed by thermal annealing. Therefore, the annealing process is recommended as the effective post-treatment process to remove these parasitic organic bonds. Finally, a fully inorganic AZO thin film was formed by the post-annealing process with a slightly decreased surface roughness, as observed above.

We investigated the optical properties of the AZO-coated glass, as shown in Fig. 3c. The transmittance spectra exhibited a noticeable difference, especially in the short-wavelength region (<600 nm), depending on the fabrication conditions of the AZO films. For instance, the average transmittance in the range from 300 to 1600 nm was 92.0, 90.3 and 87.0 for the as-grown, UV-treated, and annealed AZO thin films on glass, respectively. The transmittance of the reference quartz glass was 93.5%. We found that the post-processing induces a shift in the absorption edge of the AZO thin film to a longer wavelength. The redshift corresponds to crystallization and fewer oxygen vacancies during the UV treatment and annealing process.^28,29^ We confirmed that post-annealing was the more effective method of AZO thin-film crystallization, and that the absorption edge of the AZO showed a noticeable shift following thermal annealing, compared with UV treatment.

In the space environment, high-energy photons, such as those from UV irradiation, can damage and significantly degrade the performance of photovoltaic cells and module materials, such as encapsulants and cover glass, thereby reducing the operating lifetime of the photovoltaic modules.^4,30^ To prevent this, a cover glass with efficient UV absorption and reflection is required to ensure stable photovoltaic cell operation in the space environment. Although the average transmittance of the AZO thin film prepared with post-treatment was slightly lower than that of the as-grown AZO film, it exhibited enhanced UV reflection and absorption. We confirmed that the AZO-coated quartz prepared with the thermal annealing process exhibited enhanced light absorption (∼30% at 300 nm) and reflection (∼20% at 300 nm) in the UV region, as shown in Fig. 3d.

We investigated electron radiation-shielding performance using a simulated electron beam irradiation test on the AZO-coated glasses, depending on the post-treatment process. Electron beam irradiation was performed at an electron energy of 1.2 MeV with a fluence of 1 × 10^15^ e^−^ cm^−2^. The shielding performance under electron beam irradiation was evaluated using a B3000 radiochromic film dosimeter.^31,32^ The AZO-coated glass was placed on the film dosimeter, and electron beam irradiation was subsequently conducted. We analyzed the electron radiation-shielding performance by measuring the optical color difference of the film dosimeters used. We found that the thermally annealed AZO thin film on glass exhibited a relatively low color difference following electron radiation, with a measured dosimeter color difference of 24.6%. We attribute the enhanced shielding performance of the thermally annealed AZO thin film to its increased crystallinity and the reduced percolation pathways resulting from densification during the high-temperature annealing process.^16,33^ These results confirm that achieving high crystallinity and dense microstructure in transparent shielding thin films is essential to improve their radiation-shielding effectiveness.

In comparison, the dosimeter placed beneath the UV-treated AZO film exhibited a more severe color difference (28.8%) than the as-deposited AZO thin film (25.6%). While the UV post-treatment was effective in removing residual organic compounds and solvents, it was not effective in promoting crystallization of the AZO thin film (see Fig. 3a and b). Furthermore, UV exposure can induce amorphization and may lead to the formation of secondary phases such as ZnAl_2_O_4_ (2*θ* = ∼44.7°, see Fig. 3a), which can cause a deterioration in the electron radiation-shielding performance.^34,35^ Additionally, UV irradiation may cause discoloration of the glass substrate, which can lead to a further reduction in transparency. Therefore, UV-based post-treatment should be avoided when designing effective transparent electron radiation-shielding layers.^36^

We found that the AZO thin film on glass annealed at elevated temperatures exhibited higher crystallinity, which led to enhanced electron radiation-shielding performance. However, as shown in Fig. 3c, the AZO thin film with high crystallinity also showed a reduction in optical transmittance, which can be attributed to a decreased optical energy bandgap.^37^ This indicates a trade-off between electron radiation-shielding capability and optical transparency as a function of the crystallinity of the AZO thin film. Therefore, the annealing temperature must be carefully selected to achieve an optimal balance between these two competing properties.

**Fig. 4:**
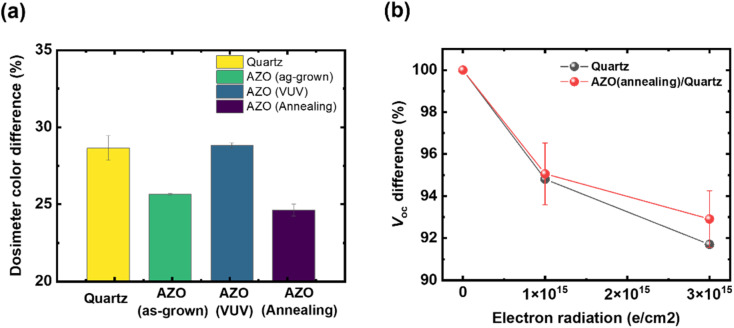
Electron radiation-shielding performance of AZO-coated quartz glass depending on post-process treatment. (a) Color difference of dosimetry with different AZO-coated glass after simulated electron radiation with an energy of 1.2 MeV and fluency of 1 × 10^15^ e^−^ cm^−2^; (b) change in *V*_oc_ under different electron beam fluences using AZO-coated glass.

We further evaluated the radiation-shielding performance of AZO-coated quartz glass using a tandem III–V photovoltaic cell (InGaP/GaAs 2J) developed in-house, which was mounted on a Si carrier wafer (see Fig. 4b and Experimental section). When bare quartz glass without the AZO coating was used as a reference shielding layer, the open-circuit voltage (*V*_oc_) of the tandem photovoltaic cell decreased by 13% after exposure to electron beam irradiation at a fluence of 3 × 10^13^ e^−^ cm^−2^. In contrast, when annealed AZO-coated quartz glass was employed, the *V*_oc_ degradation was reduced to only 8% under the same irradiation conditions. These results demonstrate that enhancing the crystallinity of the AZO layer significantly improves the radiation-shielding performance of AZO-coated glass for the tandem photovoltaic cells used in space applications.

**Fig. 5:**
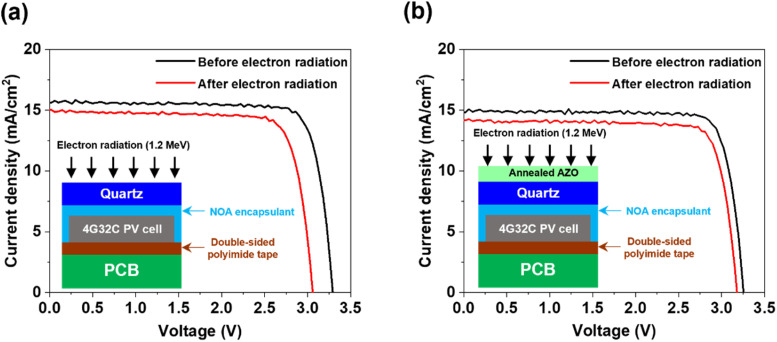
Electron radiation reliability of the space photovoltaic module with AZO-coated glass. Light *J*–*V* curves of photovoltaic modules encapsulated with (a) bare quartz glass and (b) annealed AZO-coated quartz glass using a 4G32C quadruple-junction GaAs photovoltaic cell, before and after electron beam irradiation (1.2 MeV, 1 × 10^15^ e^−^ per cm^2^). The inset shows the structure of the fabricated photovoltaic module with (a) bare quartz glass and (b) the AZO-coated quartz glass, respectively.

Finally, we fabricated a space photovoltaic module using annealed AZO-coated quartz glass and a commercially available 4G32C quadruple-junction GaAs photovoltaic cell (AlInGaP/AlInGaAs/InGaAs/Ge). To accommodate the full area of the 4G32C photovoltaic cell (30.18 cm^2^), AZO was deposited on an enlarged quartz glass substrate using an ultrasonic spray coating method, which enables scalable coating. The identical AZO precursor solution and annealing conditions described earlier were employed. The photovoltaic cell was encapsulated using the AZO-coated quartz glass on the front side and a flame-retardant 4-printed circuit board (FR4 PCB) on the rear side (see inset of Fig. 5). Further details of the space photovoltaic module fabrication process are provided in the Experimental section.

Fig. 5a and b show the light *J*–*V* curves of the photovoltaic module encapsulated with either bare quartz glass or annealed AZO-coated quartz glass under AM0 illumination conditions, both before and after electron beam irradiation (1.2 MeV, 1 × 10^15^ e^−^ cm^−2^). As shown in Fig. 5b, the photovoltaic module encapsulated with AZO-coated glass exhibited a slightly lower short-circuit current density (*J*_sc_) of 14.88 mA cm^−2^ compared to the bare quartz encapsulated one (15.65 mA cm^−2^), attributed to reduced optical transmittance of the outmost AZO layer (see Fig. 3c). Nevertheless, despite its initially lower efficiency, the AZO-coated photovoltaic module demonstrated superior radiation tolerance, maintaining higher open-circuit voltage (*V*_oc_) and fill-factor (FF) after electron irradiation (see Table 1). This improvement is attributed to the enhanced shielding effect of the thermally annealed AZO layer compared to bare quartz glass. Consequently, the photovoltaic module incorporating AZO-coated quartz glass is expected to offer an extended operational lifetime and higher reliability in harsh space environments.

**Table 1:** Performance parameters of the space photovoltaic module evaluated before and after electron beam irradiation

	Bare quartz encapsulated photovoltaic module		Annealed AZO/quartz encapsulated photovoltaic module	
	Before electron radiation	After electron radiation	Before electron radiation	After electron radiation
*V*_oc_ (V)	3.29	3.06	3.26	3.18
*J*_sc_ (mA cm^−2^)	16.65	15.03	14.88	14.18
FF (%)	82.65	80.19	83.47	82.53
Efficiency (%)	31.15	26.97	29.59	27.22

## Conclusion

In this study, we developed AZO-coated quartz glass as an effective transparent electron radiation-shielding material for the protection of space photovoltaic cells. To enhance its shielding performance, two types of post-treatment were applied: UV exposure and thermal annealing. Between them, thermal annealing was found to be more effective at removing residual organic compounds and solvents, as well as in inducing crystallization of the initially amorphous AZO thin film. The resulting thermally annealed AZO films exhibited a noticeable improvement in electron beam shielding performance due to their enhanced crystallinity and densification. The shielding effectiveness of the AZO-coated glass was further evaluated using tandem GaAs photovoltaic cells. Under electron beam irradiation conditions (1.2 MeV, 1 × 10^15^ to 3 × 10^15^ e^−^ cm^−2^), the AZO-coated samples exhibited a *V*_oc_ degradation of only −7.1%, compared to −8.3% for tandem photovoltaic cells protected with bare quartz glass. Finally, we fabricated a large-area (>30 cm^2^) space photovoltaic module using a commercial 4G32C quadruple-junction GaAs photovoltaic cell encapsulated with AZO-coated quartz glass. Under electron beam irradiation at a fluence of 3 × 10^15^ e^−^ cm^−2^, the AZO-coated module exhibited only a 2.37% degradation in power conversion efficiency, whereas the module encapsulated with bare quartz glass showed a 4.18% reduction. Although the AZO coating slightly reduced initial efficiency due to lower optical transmittance, the improved radiation shielding resulted in significantly better performance retention under electron irradiation. These results demonstrate that thermally annealed AZO-coated glass provides enhanced radiation-shielding properties and offers long-term stability for use in space photovoltaic applications.

## Experimental section

### Sample preparation (preparation of AZO thin films)

Zinc acetate dihydrate [Zn(CH_3_COO)_2_·2H_2_O] (99.5%, Sigma Aldrich) and aluminum nitrate nonahydrate [Al(NO_3_)_3_·9H_2_O] (99.5%, Alfa Aesar) were used as the sources of Zn and Al, respectively. For solution preparation, zinc acetate dihydrate was dissolved in 2-methoxyethanol (99%, Alfa Aesar) [(CH_3_)_2_CHOH] and 2–3 drops of monoethanolamine (MEA) (99%, Alfa Aesar) [H_2_NCH_2_CH_2_OH] were added to the solution as a stabilizer to enhance the stability of the solutions. The solutions were stirred at room temperature for 24 h. Before AZO coating, the quartz glass (1T) was cleaned with acetone, methanol, and DI water, sequentially, using an ultrasonic cleaner. AZO thin films were coated on the cleaned quartz glass *via* the sol–gel spin coating method. Spin coating was performed using a two-step process: first at 500 rpm for 1 s, followed by 3000 rpm for 30 s. Subsequently, the AZO-coated glass was dried at 200 °C for 5 min using a hot-plate. For the UV treatment process, the AZO-coated samples were irradiated with vacuum UV (SUS05, Ushio Inc.) at a peak wavelength of 172 nm for 40 min under ambient conditions. For the thermal annealing process, the AZO-coated samples were annealed in a box furnace at 450 °C for 30 min.

### Characterization

The surface morphology of the AZO thin film was examined using field-emission scanning electron microscopy (FE-SEM, FEI Nova230). Surface roughness (*R*_a_) was measured by atomic force microscopy (AFM, XE100, PSIA). The optical properties were analyzed using a UV-vis-NIR spectroscope (Cary 5000, Agilent Technologies). The microstructure of the AZO thin film was characterized by X-ray diffraction (XRD, Rigaku MiniFlex600) using CuKα radiation (*λ* = 1.5406 Å). Fourier-transform infrared spectroscopy (FT-IR, JASCO FT/IR-4X) was employed to analyze the chemical bonding states of the AZO thin film. To simulate electron beam irradiation conditions, an electron beam accelerator (ELV-8, EB-Tech) installed at the Korea Atomic Energy Research Institute (KAERI) was used, operating at 1.2 MeV with fluences ranging from 1 × 10^15^ to 3 × 10^15^ e^−^ cm^−2^. The radiation-shielding performance was evaluated using B3000 radiochromic film dosimeters (GEX Corporation). The color change of the irradiated dosimeters was measured using a spectrometer (Genesys 30, Thermo Scientific).

### Tandem photovoltaic cell for electron radiation experiment

The InGaP/GaAs 2J tandem photovoltaic cell structures were grown in a vertical chamber of a low-pressure metalorganic chemical vapor deposition (MOCVD) reactor. Trimethylindium (TMIn) and trimethylgallium (TMGa) were used as group III precursors, while AsH_3_ and PH_3_ were used as group V sources. SiH_4_, DEZn, CBr_4_, and DETe were employed as n-doping, p-doping, and tunnel junction doping sources. Tandem photovoltaic cell devices of 1 cm^2^ area were fabricated by photo-lithography, metal deposition, wet-etching, and back-end processes. AuGe/Ni/Au and Ti/Pt/Au multi-layers were employed as n-type and p-type ohmic contacts, respectively, and deposited by an e-beam evaporator. A 30 μm-thick Au layer was electroplated on the reverse side as a mechanical support for the epitaxial cell layers. Finally, MgF_2_/ZnS double-layer coatings were deposited on the front surface as an antireflection coating (ARC).

### Photovoltaic modulization process

A 4G32C quadruple-junction GaAs photovoltaic cell with a cell area of 30.18 cm^2^ was purchased from AZURE SPACE. Photovoltaic cell electrodes were connected with an Ag-coated Kovar interconnector *via* a parallel gap resistance welding (PGRW) method. The AZO precursor solution, as described above, was deposited on quartz glass substrates (0.4 T) using an ultrasonic spray coating system (ExactaCoat, SONOTEK Corporation) and subsequently annealed in a box furnace under identical conditions to those previously described. Norland Optical Adhesive 63 (NOA63) was dispensed onto the GaAs photovoltaic cell front surface, and the prepared AZO-coated quartz glass was placed on top. The assembly was then cured under UV (365 nm) irradiation. The resulting assembly was mounted on the FR4 PCB using double-sided polyimide adhesive film. Kovar interconnectors connected to the photovoltaic cell were soldered to the PCB terminals to complete the electrical connection.

## Conflicts of interest

The authors declare no competing financial interest.

## Data Availability

The data that support the findings of this study are available from the corresponding author upon reasonable request.
